# PINCH is an independent prognostic factor in rectal cancer patients without preoperative radiotherapy - a study in a Swedish rectal cancer trial of preoperative radiotherapy

**DOI:** 10.1186/1471-2407-12-65

**Published:** 2012-02-10

**Authors:** Annica Holmqvist, Jingfang Gao, Birgitta Holmlund, Gunnar Adell, John Carstensen, Dianne Langford, Xiao-Feng Sun

**Affiliations:** 1 Department of Medical Oncology, Institute of Clinical and Experimental Medicine, Linköping University, S-58185 Linköping, Sweden; 2 Institute of Health and Society, Faculty of Health Sciences, Linköping University, Linköping, Sweden; 3 Department of Neuroscience, Temple University School of Medicine, Philadelphia, PA, USA

**Keywords:** PINCH, Radiotherapy, Prognosis, Rectal cancer, Immunohistochemistry

## Abstract

**Background:**

The clinical significance between particularly interesting new cysteine-histidine rich protein (PINCH) expression and radiotherapy (RT) in tumours is not known. In this study, the expression of PINCH and its relationship to RT, clinical, pathological and biological factors were studied in rectal cancer patients.

**Methods:**

PINCH expression determined by immunohistochemistry was analysed at the invasive margin and inner tumour area in 137 primary rectal adenocarcinomas (72 cases without RT and 65 cases with RT). PINCH expression in colon fibroblast cell line (CCD-18 Co) was determined by western blot.

**Results:**

In patients without RT, strong PINCH expression at the invasive margin of primary tumours was related to worse survival, compared to patients with weak expression, independent of TNM stage and differentiation (*P *= 0.03). No survival relationship in patients with RT was observed (*P *= 0.64). Comparing the non-RT with RT subgroup, there was no difference in PINCH expression in primary tumours (invasive margin (*P *= 0.68)/inner tumour area (*P *= 0.49). In patients with RT, strong PINCH expression was related to a higher grade of LVD (lymphatic vessel density) (*P *= 0.01)

**Conclusions:**

PINCH expression at the invasive margin was an independent prognostic factor in patients without RT. RT does not seem to directly affect the PINCH expression.

## Background

Particularly interesting new cysteine-histidine rich protein (PINCH) is a five LIM domain protein whose gene is located on chromosome 2q12.2. PINCH is a part of the PINCH-ILK-Parvin (PIP) complex connected to integrins at the cell surface, and acts as an adapter protein for signal transduction through the cytosol [1]. The PIP complexes provide crucial physical linkages between integrins and the actin cytoskeleton and transduce signals from the extracellular matrix to intracellular effectors [2,3]. These effectors further regulate the cytoskeleton organisation, spreading, motility and proliferation of the cell [4,5]. PINCH is up-regulated in several types of cancers, and increases from normal mucosa to primary tumour to metastasis [6-9]. In previous studies of colorectal tumours, PINCH was more intensely stained at the invasive margin compared to the intratumoural stroma, and related to worse prognosis [6,7].

Few have analysed the relationship between PINCH and radiotherapy (RT). Previously, it was shown that PINCH was radio-resistant by activating Akt [10]. Others found PINCH to be similarly radio-sensitive under adherent and suspension conditions [11]. RT is known to regulate the cell cycle in the G_1_, G_2 _and S-phase and PINCH has been shown to be involved in cell cycle progression and survival [4,5,12]. Since both PINCH and RT seems to be involved in cell cycle regulation and the location of PINCH at the invasive margin seems to play an important role in patient's prognosis, we wanted to investigate the relationship between PINCH and RT, both at the invasive margin and inner tumour area, in primary rectal tumours.

The aim of this study was to investigate PINCH expression in tumours and its relationship to RT, clinical (gender, age, TNM stage, differentiation, local recurrence, distant recurrence and survival), pathological (lymph-angiogenesis, angiogenesis, inflammatory infiltration and necrosis) and biological factors (apoptosis) in rectal cancer patients with or without RT.

Immunohistochemical studies of colorectal tumours have shown that PINCH was widely expressed in the stroma around tumour cells [6,7]. Here, cell lines of normal fibroblasts were used to study the expression of PINCH and to further analyse the relationship between PINCH expression and RT.

## Methods

### Patients

This study included patients from the Southeast Swedish Health Care region who participated in a Swedish clinical trial of preoperative RT during 1987-1990 [13]. All patients were diagnosed with rectal adenocarcinoma. The present study included 137 primary tumours, where 72 patients underwent tumour resection alone and 65 patients underwent preoperative RT before surgery. RT was administered with 25 Gray (Gy) in 5 fractions during a median of 6 days (range, 5-12 days). Surgery was then performed a median of 3 days (range, 1-13 days) after RT. None of the patients received adjuvant chemotherapy before or after surgery. The mean age of the patients was 67 years (range, 36-85 years) and the median follow up was 86 months (range, 0-193 months). Additional characteristics of the patients and tumours are present in Table 1. The Research Ethics Committee professor Åke Bertler at Linkoping University hospital, number 86151, approved the study.

**Table 1:** Patient and tumour characteristic (*n *= 137)

Characteristics			
	**Non-RT *n *(%)**	**RT *n *(%)**	***P***

Gender			

Male	42 (58)	40 (62)	0.70

Female	30 (42)	25 (38)	

Age (years)			0.84

≤ 67	30 (42)	26 (42)	

> 67	42 (58)	39 (60)	

TNM			0.11

I	20 (28)	22 (34)	

IIA	18 (25)	21 (32)	

IIIA	8 (11)	1 (2)	

IIIB	11 (15)	11 (17)	

IIIC	11 (15)	4 (6)	

IV	4 (6)	6 (9)	

Differentiation			0.63

Good	2 (3)	2 (3)	

Moderate	58 (81)	48 (74)	

Poor	12 (16)	15 (23)	

Surgical type			0.17

Rectal amputation	36 (50)	25 (38)	

Anterior resection	36 (50)	40 (62)	

Resection margin			0.33

Tumor free	70 (97)	61 (94)	

Tumor	2 (3)	4 (6)	

To anal verge (cm)			

Mean	7.5	8.5	

The level of lymphangiogenesis and angiogenesis [14] were determined by immunohistochemistry. Inflammatory infiltration and necrosis were analysed on HE-stained sections [15] and apoptotic cells were detected by the terminal deoxynucleotidy transferase-mediated dUTP-biotin nick end-labelling (TUNEL) assay [16]. The data were taken from our previous studies performed at our laboratory.

### Immunohistochemistry

Five-micrometer formalin fixed, paraffin-embedded sections were deparaffinised in xylene, rehydrated with a graded series of ethanol to water. The sections were treated by high pressure cooking for 10 min with Tris-ethylenediaminetetraacetic acid (EDTA) buffer (pH 9.0) and kept at room temperature for 30 min. Following pre-incubation in methanol with 0.3% H_2_O_2 _for 20 min, the sections were incubated with protein block (Dako, Carpinteria, CA) for 10 min and then incubated with rabbit anti-PINCH antibody at 6 μg/ml in antibody diluent (Dako) for 1 h at room temperature. After washing in phosphate-buffered saline (PBS, pH 7.4), the sections were incubated with an anti-rabbit/mouse secondary antibody provided by Dako ChemMate EnVision Detection Kit (Dako) at room temperature for 25 min and washed with PBS. Subsequently, the sections were subjected to 3,3'-diaminobenzidine tetrahydrochloride for 8 min and then counterstained with hematoxylin. The positive controls were primary colorectal tumours known to stain positive for PINCH and the negative controls were primary rectal tumours where PBS was used instead of the primary antibody. In all staining procedures, the positive controls showed clear immunostaining but no immunostaining was observed in the negative controls.

The staining results of PINCH in tumours were the mean of scores by two independent authors (A. Holmqvist and J. Gao) in a blinded fashion without any knowledge of clinical and biological information.

The staining intensity was determined in 10-20 areas (depending on the size of the section) at 400 × magnification. The cases were considered to have negative, weak, moderate or strong staining. The percentage of stained cells was estimated among the total number of cells by reading 10-20 areas at 400 × magnification, regardless of the staining intensity. The cases were scored as < 25%, 25-49%, 50-75%, or > 75%, respectively. To avoid artificial effect, the cells on the margins of sections and areas with poorly presented morphology were not counted. In the cases with discrepant results in the staining score, a consensus score was reached after re-examination.

### Cell culture and radiation procedure

The CCD-18 Co cell line derived from human colon fibroblasts (ATCC, Rockville, MD), was a kind gift from Dr. R Palmqvist (Department of Pathology, Umeå University, Sweden). The cells were cultured in Dulbecco's Modified Eagles Medium (DMEM) with Glutamax™ and supplemented with 1% Penicillin-Streptomycin and 10% FBS (Invitrogen, Carlsbad, CA).

For all experiments, cells were seeded at a density of 60.000 cells/cm^2 ^and irradiated with photons from a 6 MV linear accelerator Varian Clinac 600 C/D (Varian Medical Systems, Palo Alto, CA). The field size was 30 × 30 cm and the distance between sources and cells was 100 cm. Acrylic glass plates were placed above (3 cm thick) and underneath (10 cm thick) the cells.

The cells were exposed to single doses of 0, 2, 5 or 10 Gy at room temperature. The most significant biological change in protein expression was observed with the radiation dose of 2 Gy, as also shown by previous studies [17]. Therefore 2 Gy was used for further analyses in our study. The controls (0 Gy) were handled under the same environmental conditions as the treated cells. Following radiation, cells were harvested at 8, 24, 48 and 72 h for western blot analysis. All experiments were repeated three times.

### Western blot analysis

After radiation, cells where washed in PBS and lysed in RIPA buffer, containing 150 mM NaCl 2% Triton, 0.1% SDS, 50 mM Tris pH 8.0 and a Protease Inhibitor Cocktail without chelating reagents (Sigma-Aldrich, Stockholm, Sweden). Protein concentration was determined with the colorimetric BCA protein assay reagent (Pierce, Woburn, MA). Samples containing 30 μg protein where separated by electrophoresis in a Mini-PROTEAN TGX™ precast 12% Gel (Bio-Rad, Hercules, CA) for 55 min at 200 V. The separated proteins were transferred to a PVDF-membrane (Amersham Biosience/GE Healthcare, Piscataway, NJ). The membranes were blocked with 5% non-fat dried milk in Tris-buffered saline (TBS) containing 0.1% Tween 20 (TBST) and incubated with primary PINCH antibody (REF) 1 μg/mL overnight at 4°C in TBST and 1% non-fat dried milk. The membranes were washed and incubated for 1 h at room temperature with a HRP conjugated polyclonal goat anti-mouse secondary antibody (1:5,000, Dako, Cytomation, Glostrup, Denmark) followed by enhanced chemiluminescence (ECL)(Amersham Biosiences/GE Healthcare). To verify equal loading of the wells the membranes were reincubated with a primary mouse polyclonal anti-β-actin antibody (1:5,000, Sigma-Aldrich, Steinheim, Germany) and a secondary polyclonal goat ant-mouse antibody (1:10,000, Dako, Cytomation, Glostrup, Denmark).

### Statistical analysis

The Chi-square method was used to analyse the relationship between PINCH expression in tumours and the clinical, pathological or biological factors. Cox's proportional hazard model was used to estimate the relationship between PINCH expression and survival, including both univariate and multivariate analyses. Survival curves were computed according to the Kaplan-Meier method. Tests were two-sided and *P <*0.05 was considered statistically significant.

## Results

### PINCH expression in primary tumours

**Figure 1:**
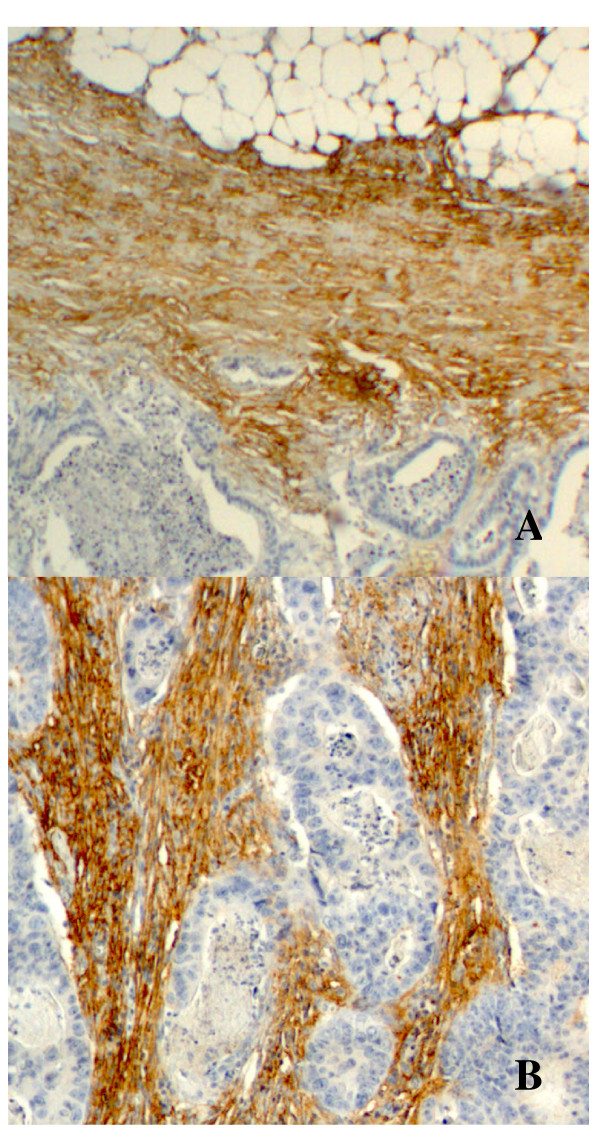
**Strong PINCH expression in tumour-associated stroma at the invasive margin (A) and inner tumour area (B) of primary rectal tumours**.

PINCH protein expression was analysed at the invasive margin (Figure 1A) and inner tumor area (Figure 1B) of 137 primary tumours. At the invasive margin 16 (12%) cases had weak PINCH expression, 51 (37%) cases had moderate and 70 (51%) cases had strong PINCH expression. At the inner tumour area 12 (9%) cases had weak expression, 66 (48%) cases moderate and 59 (43%) cases had strong PINCH expression. There were no negative cases for PINCH either at the invasive margin or inner tumour area. In this study, no statistically significant results where found when the percentage of stained cells where analysed (*P *> 0.05), therefore, further analysis only shows the result of the staining intensity.

Staining scores for PINCH in primary tumours with and without RT are presented in Table 2. For further analysis the expression levels of PINCH were classified in either a weakly stained subgroup if they were scored as negative, weak or moderately stained, or into a strong subgroup if they were strongly stained (Table 2).

**Table 2:** PINCH expression in primary rectal tumours with or without RT

	Staining intensity		
	**Weak *n *(%)**	**Strong *n *(%)**	***P***

**Invasive margin**			

Non-RT	34 (47)	38 (53)	

RT	33 (51)	32 (49)	*P *= 0.49

**Inner tumour area**			

Non-RT	39 (54)	33 (46)	

RT	39 (60)	26 (40)	*P *= 0.68

Upon comparing the PINCH expression at the invasive margin with that at the inner tumour area in the 137 primary tumours, 23 of the cases (17%) had stronger staining at the invasive margin, 99 of the cases (72%) showed the same staining levels, and 15 cases (11%) had weaker staining (*P *= 0.06).

### PINCH expression in primary tumours in relation to clinical variables

**Figure 2:**
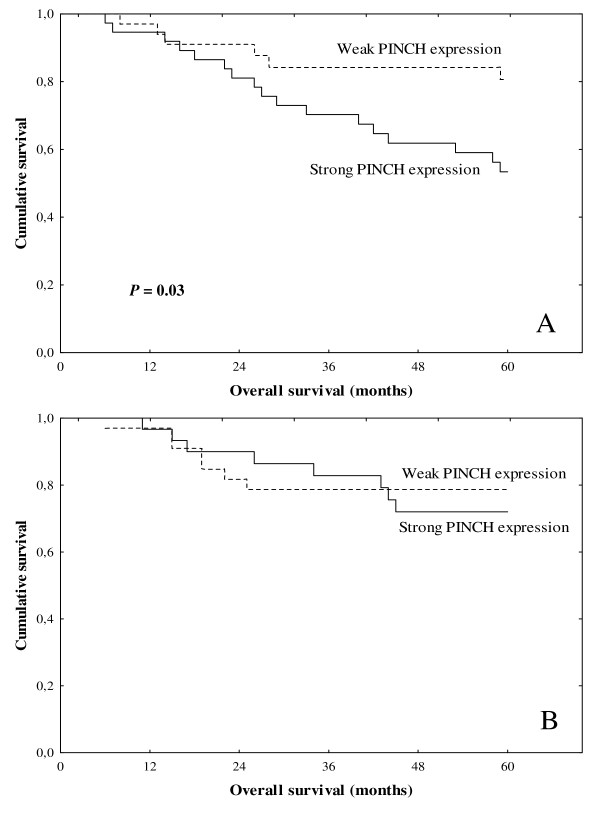
**PINCH expression at the invasive margin of primary rectal cancer in relation to survival in patients without RT (A) or with RT (B)**.

We further analysed the relationships between PINCH expression at both the invasive margin and at the inner tumour area of primary tumours with patient survival. At the invasive margin of tumours, either in all patients (*P *= 0.04) or in the non-RT group (*P *= 0.03, Figure 2A), strong expression of PINCH was related to shorter survival time, compared to those with weak PINCH expression. In the non-RT group, a further multivariate analysis showed that the prognostic significance still remained after adjusting for both TNM stage and differentiation (*P *= 0.03). In patients with RT, no significant difference was found between the expression of PINCH and survival time (*P *= 0.64, Figure 2B). A further interaction analysis between PINCH (at the invasive margin), RT and survival did not show a statistically significant result (*P *= 0.30).

When the inner tumour area was analysed, no significant relationship was present for PINCH expression and survival in patients from the non-RT or RT subgroups (*P *> 0.05).

No significant differences were found between the subgroups of non-RT and RT in PINCH expression of either at the invasive margin (*P *= 0.68) or inner tumor area (*P *= 0.49) as shown in Table 2.

There was no significant relationship of PINCH expression of either at the invasive margin or inner tumour area with local recurrence, distant recurrence or disease free survival, in the whole group of patients and in the subgroups of non-RT and RT in primary tumours (*p *> 0.05).

### PINCH expression in primary tumours in relation to pathological and biological factors

We further analysed PINCH expression of primary tumours at the invasive margin and at the inner tumour area and the relationship to clinical, pathological and biological factors.

In all patients, strong PINCH expression was related to weak inflammatory infiltration (*P *= 0.002) and a higher grade of necrosis (*P *= 0.03) at the invasive margin of tumours. In the non-RT subgroup, strong PINCH expression was related to weak inflammatory infiltration (73% of 33 cases Vs 37% of 35, *P *= 0.003) and less apoptosis (66% of 35 cases Vs 36% of 33, *P *= 0.02) and positively related to age (*P *= 0.005). There was no significant relationship in the RT group (*P *> 0.05).

**Figure 3:**
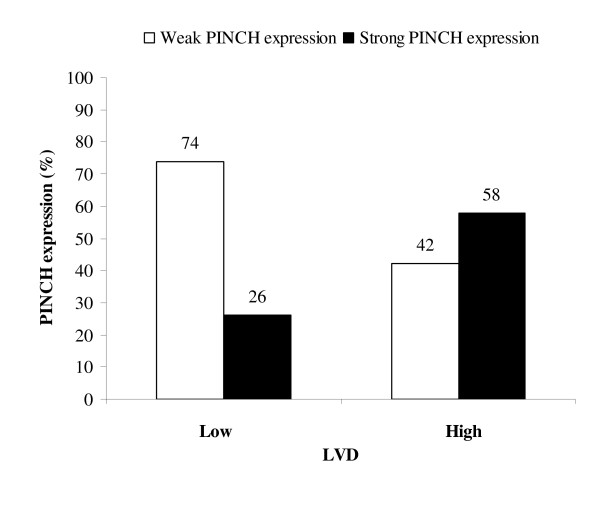
**PINCH expression at the inner tumour area of primary rectal cancer in relation to LVD in patients with RT**.

In all patients and in the non-RT subgroup, strong PINCH expression at the inner tumour area was related to a higher blood vessel density (BVD) located at the periphery (*P *= 0.03, *P *= 0.02) and weak inflammatory infiltration (*P *= 0.0005, *P *= 0.0007). In the RT group, strong PINCH expression was related to a higher grade of LVD (*P *= 0.01, Figure 3) and more necrosis (*P *= 0.01).

### PINCH expression in CCD-18 co with or without RT

**Figure 4:**
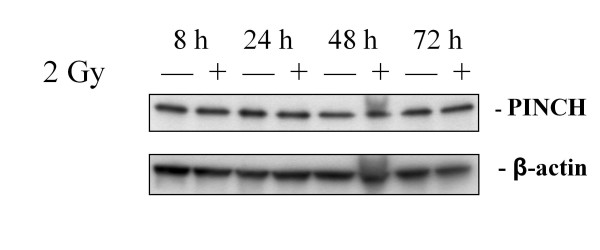
**PINCH and β-actin expression in CCD-18 Co cells without (-) or with RT (+), analysed 8 h, 24 h, 48 h and 72 h after RT**.

The protein expression of PINCH in CCD-18 Co cells was studied without (0 Gy) and with RT (2 Gy), and analysed over time at 8, 24, 48 and 72 h after RT. The expression of PINCH in the CCD-18 Co cells showed equally thick single clear bands as shown in Figure 4. No differences were observed in PINCH protein expression between cells without RT (-, 0 Gy) or with RT (+, 2 Gy) harvested at different times.

## Discussion

This is the first study of PINCH expression in rectal cancer patients who participated in a Swedish clinical trial of preoperative RT. In patients without RT, strong PINCH expression at the invasive margin of primary tumours was related to worse survival compared to patients with weak PINCH expression. The prognostic significance still remained even after adjustment for both TNM stage and differentiation. This result was in line with others who studied PINCH expression in 174 colorectal cancer patients [7]. After RT, there was no relationship between PINCH and survival. A further interaction analysis showed no statistically significant result, which might indicate that the number of deaths in the RT group was low. As far as we know, this is the first study of PINCH in relation to RT in patients. A previous cell line study of mouse embryonic fibroblasts and human colon, lung, cervix, skin and pancreas tumours showed that PINCH was radio-resistant by activating Akt1 [10]. Others showed that the radio sensitivity in PINCH depleted normal and malignant cells was similar under adherent and suspension conditions [11]. In this study, we did not found any significant difference in PINCH expression between the subgroups of non-RT and RT, which might be explained by a too short interval between RT and surgery. The best clinical effects of RT on tumour tissue are known to be received around 5 weeks after RT. In our study, the patients received preoperative RT and went through surgery within 1-13 days after RT, which might be a too short time to receive the optimal clinical effect by RT. Even though there was no significant difference in PINCH expression between the non-RT and RT subgroups, the survival for patients with weak and strong PINCH expression seemed to change with RT, which makes us suggest that PINCH might not be directly increased by RT, but maybe activated by RT via other biological pathways.

PINCH together with its binding partners are known to regulate cell survival and apoptosis [4,10,12]. Previously, it was shown that PINCH induced radio-resistance by activating Akt1 via PP1α [10]. Others showed that an inhibition of the PINCH-ILK complex increased apoptosis by reducing the activity of protein kinase B (PKB)/Akt in immortalised HeLa cells [4].

The epidermal growth factor (EGF) is a well-known growth factor, which is mitogenic and stimulates cell division by binding to a tyrosine kinase receptor on the cell membrane. RT is known to up-regulate the EGF receptor [18,19]. Recently, it was shown that EGF together with its receptor was associated with PINCH via the adaptor protein Nck-2 [4]. RT induced cell damage might increase the production of EGF witch further activates PINCH via Nck-2. We suggest that the cell damage that RT causes could be the initiating mechanism, not for an up-regulation of PINCH, but for an activation of PINCH, via the PKB/Akt pathway or EGF and Nck-2 pathway.

The lymphatic vasculature drains interstitial fluid from tissue and is one of the most common ways for tumour cells to metastasis and spread. Recently, it was shown that LVD was increased by RT [20]. In the present study of the patients with RT, a positive relationship was found between PINCH at the inner tumour area and LVD. We suggest that PINCH might stimulate the production of new lymph vessels as a reaction to RT induced cell damage. The positive relationship between PINCH and LVD after RT, might increase the area for potential escape of tumour cells into the lymphatic circulation.

In line with our previous findings, our present study of the fibroblast cell line showed no changes in PINCH expression after RT. PINCH are known to be widely expressed in fibroblasts and increases from normal mucosa to tumour [6-9]. The cells used in our study are supposed to be normal fibroblasts with less expression of PINCH than the fibroblasts in the tumour tissue. Since we did not find any differences in PINCH expression in tumours after RT, the probability to find differences in PINCH expression in the fibroblast cells after RT might be low.

The infiltration of inflammatory cells in tumour tissue is considered as an important factor of the host response, and is related to improved survival in colorectal cancer [14]. A recent study on colorectal cancer patients showed that a high PINCH expression was related to weak inflammatory infiltration [7]. In line with this study, at the inner tumor area and invasive margin, in all patients and in the non-RT subgroup, we found a relationship between strong PINCH expression and weak inflammatory infiltration. Gao et al. (2004) showed an increased amount of PINCH in myofibroblasts suggesting that these cells induce the tumour reaction against inflammatory cell infiltration.

Apoptosis is programmed cell death and decreased apoptosis are related to worse survival in tumours. Previously, it was shown that PINCH inactivated the intrinsic apoptotic pathway [12]. Ours found the same result in non-irradiated patients, where a strong PINCH expression at the invasive margin was related to less apoptosis. These findings strengthen our previous relationships between strong PINCH expression and less inflammatory infiltration and worse survival. We suggest that PINCH at the invasive margin might facilitate tumour progression and survival by inhibiting inflammatory infiltration and reduce apoptosis.

The relationship between BVD and survival has been studied extensively. In a previous study by ours on the same series of the cases used in the present study, patients in the non-RT subgroup with BVD at the periphery tended to have a worse outcome than the patients with BVD at the inner tumour area/invasive margin [14]. In the present study of all patients and in patients without RT, PINCH expression at the inner tumour area was related to a higher BVD at the periphery of tumours. At the periphery, PINCH might create an environment that makes it easier for tumour cells to transit into the blood system.

Tumour necrosis is caused by a rapid tumor growth without sufficient blood supply, which leads to ischemia and necrosis of the tumour cells. Previous studies by others did demonstrate that necrosis was associated with a poor clinical outcome [21]. In the present study of all patients, we found a positive relationship between PINCH at the invasive margin and necrosis. Since PINCH is involved in cell regeneration we suggest that PINCH might induce cell proliferation which further leads to un-sufficient blood supply, ischemia and necrosis of the tumour tissue.

In a previous study by ours on the same series of the cases used in the present study, necrosis was increased by RT [15]. In the present study, in patients with RT, a strong PINCH expression was positively related to more necrosis. PINCH seems to be involved in the initiation of necrosis induced either by rapid tumour growth or by RT.

In this material the surgery was performed either by anterior resection or rectum amputation. In the 1980th a new surgical technique called total mesorectal excision (TME) was introduced, which was shown to reduce the risk of local recurrence up to 11% and increased the overall survival up to 40%. The combination of preoperative RT with TME further reduced the local recurrence rate up to around 5% [22]. Even though the local recurrence rate has been reduced the mortality rate is still high (40-50%) and there are still huge variations in response to preoperative RT in patients with the same tumour stage. Therefore it is of great importance to identify good predictive and prognostic factors such as PINCH in order to select the best suited patients for preoperative RT in the future.

## Conclusion

PINCH expression at the invasive margin was an independent prognostic factor in patients without RT, but not in the patients with RT. After RT, PINCH expression was unchanged, suggesting that PINCH might not be directly increased by RT. The positive relationship between PINCH and LVD after RT, might potentiate the area for tumour cells to escape into the lymphatic system. This issue needs to be addressed on a larger series of cases. An expanded cell culture study is planned to further investigate the relationship between PINCH and RT.

## Competing interests

The authors declare that they have no competing interests.

## Authors' contributions

AH carried out the study design, implementation of both the immunohistochemical and cell culturing procedure, interpretation of the immunohistochemical and cell culture results, statistical analysis and preparation of the article for publication. JFG participated in the immunohistochemical interpretation. BH participated in the implementation of the cell culturing procedure and interpretation of the cell culture results. GA participated in the data acquisition and study design. JC participated in the statistical analysis, quality control of data and algorithms. DL provided us with antibodies for both immunohistochemical and cell culture analysis and helped us to draft the manuscript. X-FS participated in the study design, data analysis, and interpretation of the study results and the preparation of the article for publication. All authors have read and approved the final draft.

## Pre-publication history

The pre-publication history for this paper can be accessed here:

http://www.biomedcentral.com/1471-2407/12/65/prepub
